# Enteropathogenic E. coli, Salmonella, and Shigella: masters of host cell cytoskeletal exploitation.

**DOI:** 10.3201/eid0502.990205

**Published:** 1999

**Authors:** D L Goosney, D G Knoechel, B B Finlay

**Affiliations:** University of British Columbia, Vancouver, British Columbia, Canada.

## Abstract

Bacterial pathogens have evolved numerous strategies to exploit their host's cellular processes so that they can survive and persist. Often, a bacterium must adhere very tightly to the cells and mediate its effects extracellularly, or it must find a way to invade the host's cells and survive intracellularly. In either case, the pathogen hijacks the host's cytoskeleton. The cytoskeleton provides a flexible framework for the cell and is involved in mediating numerous cellular functions, from cell shape and structure to programmed cell death. Altering the host cytoskeleton is crucial for mediating pathogen adherence, invasion, and intracellular locomotion. We highlight recent advances in the pathogenesis of enteropathogenic Escherichia coli, Salmonella Typhimurium, and Shigella flexneri. Each illustrates how bacterial pathogens can exert dramatic effects on the host cytoskeleton.


### Enteropathogenic *E. coli*, *Salmonella*, and *Shigella*: Masters of Host Cell Cytoskeletal Exploitation

**Danika L. Goosney, Derek G. Knoechel, and B. Brett Finlay**
University of British Columbia, Vancouver, British Columbia, Canada

Bacterial pathogens have evolved numerous strategies to exploit their host's cellular processes so that they can survive and persist. Often, a bacterium must adhere very tightly to the cells and mediate its effects extracellularly, or it must find a way to invade the host's cells and survive intracellularly. In either case, the pathogen hijacks the host's cytoskeleton. The cytoskeleton provides a flexible framework for the cell and is involved in mediating numerous cellular functions, from cell shape and structure to programmed cell death. Altering the host cytoskeleton is crucial for mediating pathogen adherence, invasion, and intracellular locomotion. We highlight recent advances in the pathogenesis of enteropathogenic *Escherichia coli, Salmonella* Typhimurium, and *Shigella flexneri*. Each illustrates how bacterial pathogens can exert dramatic effects on the host cytoskeleton.

### Enteropathogenic *Escherichia coli* (EPEC): A Model for Studying Bacterial Attachment and Effacement

Pathogenic *E. coli* strains remain a leading cause of severe and persistent infant diarrhea in developing countries. Although EPEC is recognized as a major diarrheal pathogen, until recently our understanding of how it causes disease lagged behind that of other pathogenic *E. coli*, such as enterotoxigenic *E. coli* or enteroinvasive *E. coli.*

EPEC is one of a class of pathogens identified as causing attaching and effacing (A/E) lesions on intestinal cells (1). A/E pathogens typically reside on a pedestal on the surface of the host epithelial cell and ultimately cause severe disruption of the microvilli brush border (Figure 1A). Other pathogens displaying similar histopathologic features include *Hafnia alvei*, *Citrobacter rodentium* (formerly *C. freundii* biotype 4280), and enterohemorrhagic *E. coli,* the causative agent of hemolytic uremic syndrome.

Address for correspondence: B. Brett Finlay, Biotechnology Laboratory and the Departments of Microbiology and Immunology and Biochemistry and Molecular Biology, University of British Columbia, Vancouver, British Columbia, Canada V6T 1Z3; fax: 604-822-9830; e-mail: bfinlay@unixg.ubc.ca.

### Bacterial Factors Involved in EPEC-Induced A/E Lesion Formation

The interactions between EPEC and host cells have been divided into three stages. Initial adherence to cultured epithelial cells is mediated by the formation of type IV fimbriae known as bundle forming pili (BFP) (2). While not essential for forming the characteristic A/E lesions, initial adherence helps bring the bacteria in close contact with the host cell. BFP mediate bacterial-bacterial interactions in a human intestinal organ culture model (3).

All the genes necessary for the formation of A/E lesions by EPEC are contained within a 35-kbp pathogenicity island termed the locus of enterocyte effacement (LEE) (Figure 1B) (4,5). These include the *esps* (*E. coli*-secreted protein), *esc*s (*E. coli* secretion), *sep* (secretion of *E. coli* proteins), *eae* (*E. coli* attaching and effacing that encodes intimin), and *tir* (translocated intimin receptor) genes (6).

The second stage of EPEC pathogenesis involves the secretion of bacterial proteins, some into the host cell, including EspA, EspB, and EspD (7,8). The expression of these proteins is maximal at the host body temperature (9) and at conditions similar to those found in the gastrointestinal tract (10), which implies that they may be involved in virulence. The translocation of these proteins is essential for

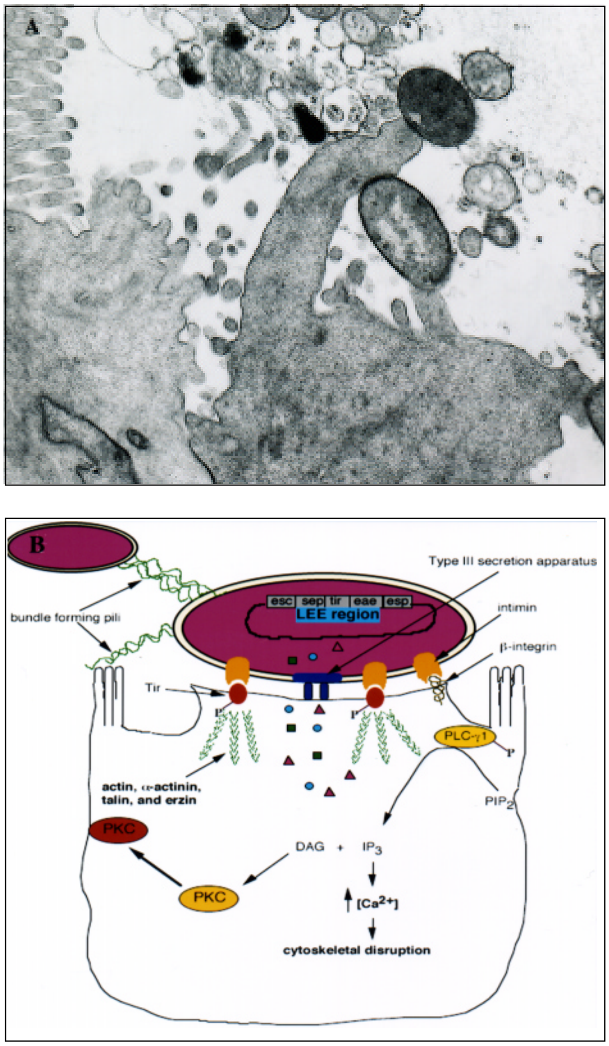

Figure 1. A. Transmission electron micrograph of an A/E lesion formed by rabbit enteropathogenic *Escherichia coli* (EPEC) infecting rabbit intestinal epithelial cells (micrograph provided by Dr. Ursula Heczko, Biotechnology Laboratory, University of British Columbia). B. Effects of EPEC infection on host intestinal epithelial cells. EPEC initially adheres to the host cell by its bundle-forming pili, which also mediate bacterial aggregation. Following initial attachment, EPEC secretes several virulence factors by a type III-secretion system. Signal transduction events occur within the host, including activation of phospholipase C (PLC) and protein kinase C (PKC), inositol triphosphate ($IP_3$) fluxes, and $Ca^{2+}$ release from internal stores. The bacterium intimately adheres to the cell by secreting its own receptor, Tir, into the host and binding to it with its outer membrane ligand, intimin. Intimin can also bind ß1-integrins. Several cytoskeletal proteins are recruited to the site of EPEC attachment, including actin, α-actinin, talin, and ezrin. Cytoskeletal rearrangements occur following Tir-intimin binding, resulting in the formation of a pedestal-like structure upon which the pathogen resides.

activating a number of signal transduction pathways (7), although their precise role in pathogenesis is not well defined. EspA makes filamentous appendages outside the bacterium and may be part of the translocation machinery involved in delivering other virulence proteins (11). EspB is translocated into the host cytosol and membrane, where it may effect changes in the host cell's signaling pathways (12). All of these effector proteins are secreted by a type-III secretion system encoded by the *esc* and *sep* genes (6). Type-III secretion systems also play an important role in other gram-negative pathogenic bacteria such as *Yersinia*, enabling virulence factors to be translocated directly from the bacterial cytoplasm to the host-cell membrane or cytoplasm (13).

The third stage of EPEC infection is characterized as intimate attachment with the host cell. Intimin, a 94-kDa outer membrane protein encoded by the *eae* gene (14), binds to a 90-kDa tyrosine phosphorylated protein in the host membrane (15). This receptor, originally thought to be a host protein, has recently been found to be of bacterial origin and has been designated as the translocated intimin receptor (Tir) (16). As the name suggests, Tir is translocated from the bacterial cell into the host membrane, where it becomes phosphorylated on one or more tyrosine residues and functions as a receptor for its binding partner, intimin. The resultant tight association is accompanied by the formation of actin pedestals up to 10 µm in length (15). Purified intimin also binds ß1 integrins, which suggests that intimin may be binding more than one receptor on the epithelial cell (17). Although integrins are not present on the apical surface of enterocytes, they are located on the apical surface of microfold cells found in Peyer's patches along the intestinal lumen (18).

### Host-Cell Factors Involved in A/E Formation

The host cell undergoes a number of changes during infection by EPEC (Figure 1B). The most striking change in the cellular structure of the host cell is the formation of characteristic actin pedestals. Within 3 hours of infection by EPEC, host-cell actin, α-actinin, talin, erzin, and villin accumulate directly under the bacteria (19,20). The latter four cytoskeletal components are involved in cross-linking of actin microfilaments. Localized actin accumulation is so distinct that it forms the basis of an in vitro diagnostic test for EPEC, which uses fluorescein-tagged phalloidin to detect actin accumulation within infected cells (21). The actin pedestals are not static; instead they lengthen and shorten, resulting in apparent movement of EPEC along the host-cell surface (20). The pedestals resemble microvilli in the distribution of actin and villin (20). Microtubule and intermediate filament structures are not affected by EPEC virulence factors (19).

Intracellular calcium levels also seem to play a role in EPEC pathogenesis. EPEC-infected HEp-2 cells show significant elevation of intracellular calcium levels (22), and buffering of these levels can prevent or delay the formation of A/E lesions (23). Increases in intracellular calcium levels can result in the depolymerization of actin by villin (a calcium-dependent microvillus protein) and a breakdown of the host cytoskeleton not unlike that seen in EPEC-infected cells (24). Inositol triphosphate ($IP_3$) is involved in the release of $Ca^{2+}$ from intracellular stores, and increased levels of $IP_3$ (25) and inositol phosphate fluxes (26) have been observed in EPEC-infected cells. EPEC interactions with PLC-γ1 HeLa epithelial cells activate a number of proteins, including phospholipase C-γ1 (PLC-γ1) (27). Phosphorylation of PLC-γ1 leads to the $IP_3$ and $Ca^{2+}$ fluxes mentioned above, underscoring the importance of this signaling event. Cytosolic protein kinase C also gets activated upon EPEC infection and translocates to the plasma membrane (28).

Despite the dramatic changes induced by EPEC in the cytoskeleton, there appears to be little involvement of the Rho family of small GTP-binding proteins normally involved in cytoskeletal rearrangements (29). Inhibition of Rho, Rac, and Cdc42 by compactin and *Clostridium difficile* ToxB, as well as dominant negative alleles, had no effect on pedestal formation by EPEC, which suggests that this pathogen uses a nontraditional mechanism to rearrange actin.

### *Salmonella* Typhimurium: A Model for Studying Bacterial Invasion

*S.* Typhimurium is a gram-negative bacterium that causes a variety of diseases, from gastroenteritis in humans to typhoid fever in mice. *S.* Typhimurium infections are contracted by oral ingestion and penetration into the intestinal epithelium before induction of systemic (invasive) disease. Invasion into the host intestinal cells results in dramatic morphologic changes to the cell that are due to exploitation of the host cytoskeleton.

Once in close contact with the epithelium, *Salmonella* induces degeneration of enterocyte microvilli (30). Loss in microvillar structure is followed by profound membrane ruffling localized to the area of bacterial-host cell contact (Figure 2A) (29-31). Membrane ruffling is accompanied by profuse macropinocytosis, which leads to the internalization of bacteria into the host cells (32). The entire process occurs within minutes and when completed, *Salmonella* resides within membrane-bound vesicles, and the cytoskeleton returns to its normal distribution (33).

### Bacterial Factors Involved in *Salmonella* Invasion

*Salmonella* entry into nonphagocytic epithelial cells requires several chromosomal genes (*inv/spa*) clustered in a pathogenicity island termed SPI1 (*Salmonella* pathogenicity island 1) (34). Like EPEC, SPI1 encodes a type III-secretion system and several potential virulence factors secreted by this machinery. The type III-secretion system is activated upon host-cell contact and allows export of virulence determinants directly into the host cell, where they effect bacterial uptake (35,36). Recently, SptP, a bacterial protein encoded within SPI1, has been shown to be translocated into the host epithelial cell, where it modulates the host actin cytoskeleton through its tyrosine phosphatase activity (37) (Figure 2B). Disruption of a critical Cys residue in the catalytic domain of SptP results in loss of phosphatase activity (38). It is hypothesized that SptP may function in disrupting host actin stress fibers, thereby facilitating membrane ruffling and subsequent bacterial uptake into host cells.

Other bacterial factors are not encoded next to the secretion apparatus but instead on the genome of a cryptic bacteriophage found in the *Salmonella* chromosome. Recently, a virulence factor encoded within this genome, SopE, has been shown to be required for efficient bacterial entry into host cells (39). SopE requires the type III-secretion system to be translocated into the host cell, where it can directly stimulate actin cytoskeletal rearrangements. It acts as a guanidine exchange factor for members of the

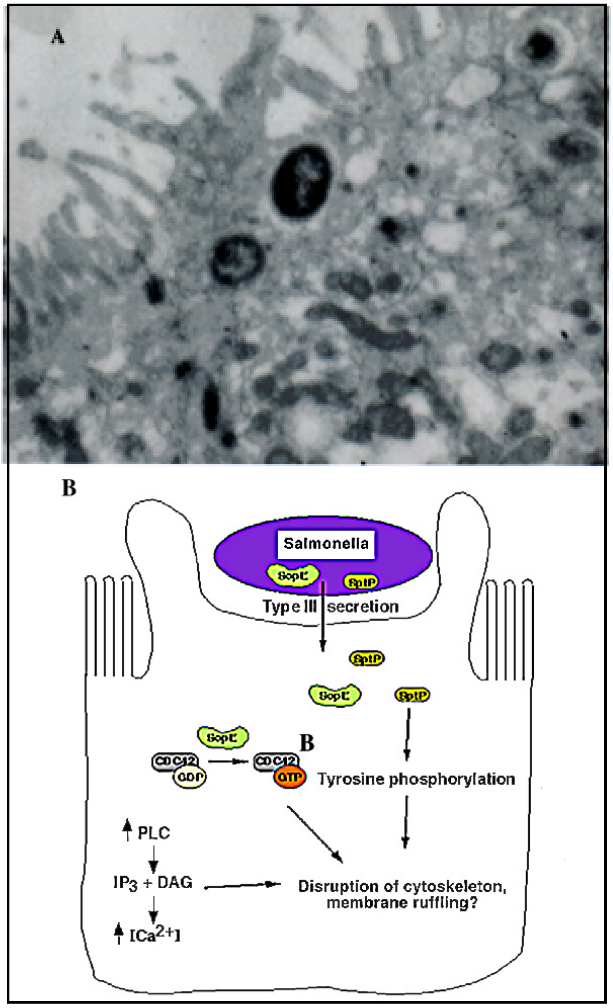

Figure 2. A. Transmission electron micrograph of *Salmonella*-induced membrane ruffling in polarized Caco-2 epithelial cells. B. *Salmonella* invasion into host epithelial cells. *Salmonella* secrete virulence proteins, including SopE and SptP, into host cells by the type III-secretion system. SopE functions as a guanidine exchange factor for small GTP-binding proteins, probably mediating the exchange of GDP for GTP on a Rho subfamily member, CDC42. SptP is a tyrosine phosphatase required for invasion, probably by disrupting the cytoskeleton. Invasion also stimulates phospholipase C (PLC) activity, leading to inositol triphosphate ($IP_3$) and $Ca^{2+}$ fluxes, which in turn may be involved in cytoskeletal rearrangements leading to membrane ruffling and *Salmonella* internalization.

Rho subfamily of small GTPases. *sopE* mutants exhibit less extensive actin cytoskeletal rearrangements upon entry into epithelial cells than do wild-type *Salmonella* (40). This discovery clearly illustrates how pathogens (which contain no primary sequence homology with host proteins) can craftily subvert the host's own signaling machinery within the cell by mimicking host proteins.

### Host Factors Involved in *Salmonella* Invasion

The massive restructuring of the host cytoskeletal components during *Salmonella* entry requires many host factors. A Rho subfamily member, Cdc42, is needed for mediating bacterial uptake through membrane ruffling (41). It is believed that the guanidine exchange activity of SopE is responsible for the stimulation of Cdc42 in the host. The pathogen also activates host PLC upon bacterial contact, leading to the production of two second messengers, which further initiate signaling events (42). As a consequence, the host cell's $Ca^{2+}$ levels are altered to trigger cytoskeletal rearrangements resulting in *Salmonella* invasion. Although EPEC and *Salmonella* use some of the same signaling components (PLC, $Ca^{2+}$ fluxes), the cytoskeletal changes induced in the host cell by each pathogen are quite different. This could be the result of different upstream or downstream effectors in the signaling pathway. Several cytoskeletal components involved in invasion have been identified. These include α-actinin, tropomyosin, ezrin, and talin (19). The specific roles of these proteins in *Salmonella* invasion are not defined.

### *Shigella flexneri*: A Model for Intracellular Motility

*S. flexneri,* a gram-negative bacillus that causes bacillary dysentery in humans, directs its own uptake into the colonic mucosa through membrane ruffling and macropinocytosis in a manner similar to *Salmonella* uptake (43,44). After engulfment, the pathogen is surrounded by a membrane-bound vacuole within the host. Unlike *Salmonella*, however, *Shigella* rapidly lyses the surrounding vacuole and is released into the cytosol, where it grows and divides (45). Once the microbe has escaped from the vacuole, it quickly becomes coated with filamentous actin and ultimately forms an actin tail at one pole of the bacterium (Figure 3A) (46,47). This actin polymerization propels the bacterium through the cytoplasm at speeds reaching 0.4 µm/sec (48). When the pathogen reaches the plasma membrane of the cell, it forms a long protrusion

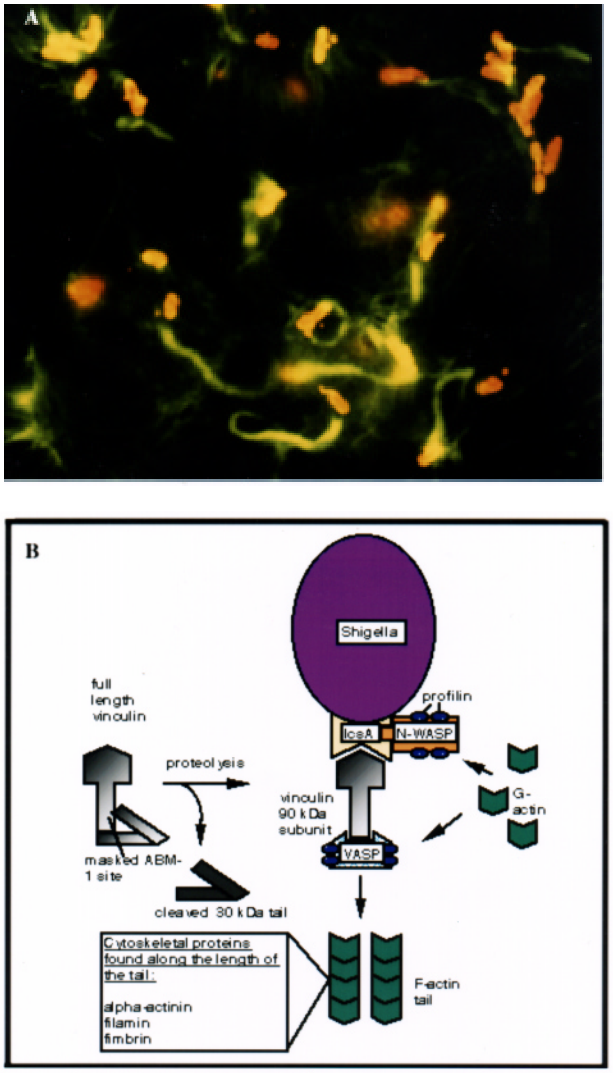

Figure 3. A. Immunofluorescence micrograph showing *Shigella* (red) propelling itself through the cytoplasm by polymerizing actin (green) (Philippe Sansonetti, Institut Pasteur, reprinted with permission from Trends in Microbiology, 1996). B. *Shigella*-mediated cytoskeletal rearrangements. The outer membrane protein, IcsA, is sufficient to drive actin-based motility of *Shigella*. IcsA directly binds two proteins, vinculin and neural-Wiskott-Aldrich Syndrome protein (N-WASP). Vinculin undergoes proteolysis within the host cell upon *Shigella* infection, producing a 90-kDa fragment that can bind to IcsA and to the vasodilator-stimulated phosphoprotein (VASP). VASP in turn can recruit profilin to the bacterial surface, which can provide actin for tail construction. N-WASP binding of IcsA can also recruit profilin to the bacterial surface and may be another means of obtaining monomeric actin for tail formation and subsequent bacterial motility.

into the neighboring cell, which subsequently internalizes the microbe (49). The bacterium again breaks out of the vacuole, thereby starting a new cycle of infection in a new host cell (50). This process allows *Shigella* to move from cell to cell without ever contacting the extracellular milieu.

### Bacterial Factors Involved in *Shigella* Motility

Analysis of mutants deficient in intracellular motility and cell-to-cell spread has identified a bacterial gene, *icsA*, necessary for *Shigella* locomotion (46,51,52). IcsA (also called VirG) is a 120-kDa outer membrane protein that hydrolyzes ATP and is localized to one pole of the bacterium, at the junction between the microbe and the actin tail (Figure 3B) (53). IcsA expression on the surface of *Shigella* is sufficient to direct actin-based motility (54,55). In fact, *E. coli* expressing IcsA can synthesize actin tails in cytoplasmic extracts (54,55).

During infection, IcsA is also detected as a 95-kDa amino-terminal fragment of the 120-kDa full-length protein (53). This proteolytic cleavage of IcsA is due to a bacterial protease, SopA (IcsP) (56,57). Cleavage is required for polarized distribution of IcsA on the bacterial surface and for proper actin-based motility of *Shigella* in infected cells (56-58).

### Host Factors Involved in *Shigella* Motility

IcsA expression on the *Shigella* surface promotes rapid accumulation of actin around the bacterium. Following bacterial division and IcsA polarization, actin tails begin to form on one end of the bacterium. Several host cytoskeletal proteins are involved in tail formation, including α-actinin (48), filamin (59), fimbrin (59), vasodilator-stimulated phosphoprotein (VASP) (60), vinculin (49,61), and neural-Wiskott-Aldrich syndrome protein (N-WASP) (63). Of these proteins, only vinculin and N-WASP are able to directly bind IcsA (61,62).

*Shigella* infection results in the cleavage of intact vinculin (120 kDa) to produce a 90-kDa fragment (63). This proteolysis unmasks an actin-based motility 1 site on vinculin, which contains a polyproline region capable of binding VASP. VASP recruitment to the bacterial surface in turn allows the recruitment of other cytoskeletal proteins, such as actin and profilin, and forms the basis of an actin-based motor for *Shigella* movement.

Recently, N-WASP was shown to be required for *Shigella* motility (62); like vinculin, it can bind IcsA directly. It is possible that N-WASP, in addition to VASP, can recruit profilin and actin to the surface of *Shigella*, thereby mediating actin polymerization. Furthermore, N-WASP contains an actin depolymerization factor/cofilin homologous region, which could be used for severing actin filaments at the pointed ends and increasing the monomeric actin concentration. The precise mechanisms involved in *Shigella*-driven actin polymerization, however, are unclear.

### Conclusions

Bacterial pathogens have evolved several mechanisms to hijack host-cell signaling machinery and disrupt the cytoskeleton. EPEC mediates its effects on the host cell from the cellular surface. It secretes its own receptor, Tir, into the host and then binds intimately to it by its outer membrane protein, intimin. Tir-intimin binding results in a dramatic reorganization of the cytoskeleton to form the pedestal upon which EPEC resides. *Salmonella*, on the other hand, actively invades intestinal epithelial cells by inducing membrane ruffling and macropinocytosis. Invasion is dependent on the secretion of virulence proteins, including SptP and SopE, into the host cell, and mediates its effects on the host from within a membrane-bound vesicle. *Shigella* is also an invasive pathogen but lyses the phagocytic vacuole and initiates intracellular actin-based locomotion to spread from cell to cell in the cytoplasm. This motility is dependent on the bacterial outer membrane protein IcsA, which recruits several actin-associated proteins to the bacterial surface. Despite the outward differences between each mode of pathogenesis, EPEC, *Salmonella*, and *Shigella* have effectively managed to subvert the host cytoskeleton for their own purposes and cause substantial diarrheal disease.

### Acknowledgments

We thank Ursula Heczko for providing the T.E.M. of rabbit EPEC pedestals.

This work was supported by Natural Sciences and Engineering Research Council of Canada postgraduate scholarships to D.L.G. and D.K. and operating grants from the Medical Research Council of Canada and a Howard Hughes International Scholar award to B.B.F.

Danika Goosney is a Ph.D. candidate in Dr. B. Brett Finlay's laboratory in the Department of Microbiology and Immunology and the Biotechnology Laboratory at the University of British Columbia. She is currently investigating EPEC-induced cytoskeletal rearrangements in cultured epithelial cells.

### References

1. Moon HW, Whipp SC, Argenzio RA, Levine MM, Giannella RA. Attaching and effacing activities of rabbit and human enteropathogenic *Escherichia coli* in pig and rabbit intestines. Infect Immun 1983;41:1340-51.
2. Giron JA, Ho AS, Schoolnik GK. An inducible bundle-forming pilus of enteropathogenic *Escherichia coli*. Science 1991;254:710-3.
3. Hicks S, Frankel G, Kaper J, Dougan G, Philips AD. Role of intimin and bundle-forming pili in enteropathogenic *Escherichia coli* adhesion to pediatric intestinal tissue in vivo. Infect Immun 1998;66:1570-8.
4. McDaniel TK, Jarvis KG, Donnenberg MS, Kaper JB. A genetic locus of enterocyte effacement conserved among diverse enterobacterial pathogens. Proc Natl Acad Sci U S A 1995;92:1664-8.
5. McDaniel TK, Kaper JB. A cloned pathogenicity island from enteropathogenic *Escherichia coli* confers the attaching and effacing phenotype on *E. coli* K-12. Mol Microbiol 1997;23:399-407.
6. Elliott SJ, Wainwright LA, McDaniel TK, Jarvis KG, Deng YK, Lai LC, et al. The complete sequence of the locus of enterocyte effacement (LEE) from enteropathogenic *Escherichia coli* E2348/69. Mol Microbiol 1998;28:1-4.
7. Kenny B, Finlay BB. Protein secretion by enteropathogenic *Escherichia coli* is essential for transducing signals to epithelial cells. Proc Natl Acad Sci U S A 1995;92:7991-5.
8. Lai LC, Wainwright LA, Stone KD, Donnenberg MS. A third secreted protein that is encoded by the enteropathogenic *Escherichia coli* pathogenicity island is required for transduction of signals and for attaching and effacing activities in host cells. Infect Immun 1997;65:2211-7.
9. Abe A, Kenny B, Stein M, Finlay BB. Characterization of two virulence proteins secreted by rabbit enteropathogenic *Escherichia coli*, EspA and EspB, whose maximal expression is sensitive to host body temperature. Infect Immun 1997;65:3547-55.
10. Kenny B, Abe A, Stein M, Finlay BB. Enteropathogenic *Escherichia coli* protein secretion is induced in response to conditions similar to those in the gastrointestinal tract. Infect Immun 1997;65:2606-12.
11. Knutton S, Rosenshine I, Pallen MJ, Nisan I, Neves BC, Bain C, et al. A novel EspA-associated surface organelle of enteropathogenic *Escherichia coli* involved in protein translocation into epithelial cells. EMBO J 1998;17:2166-76.
12. Wolff C, Nisan I, Hanski E, Frankel G, Rosenshine I. Protein translocation into host epithelial cells by infecting enteropathogenic *Escherichia coli*. Mol Microbiol 1998;28:143-55.

13. Hueck CJ. Type III protein secretion systems in bacterial pathogens of animals and plants. Microbiol Mol Biol Rev 1998;62:379 ff.
14. Jerse AE, Kaper JB. The eae gene of enteropathogenic *Escherichia coli* encodes a 94-kilodalton membrane protein, the expression of which is influenced by the EAF plasmid. Infect Immun 1991;59:4302-9.
15. Rosenshine I, Rusckowski S, Stein M, Reinscheid DJ, Mills SD, Finlay BB. A pathogenic bacterium triggers epithelial signals to form a functional bacterial receptor that mediates actin pseudopod formation. EMBO J 1996;15:2613-24.
16. Kenny B, DeVinney RD, Stein M, Reinscheid DJ, Frey EA, Finlay BB. Enteropathogenic *E. coli* (EPEC) transfers its receptor for intimate adherence into mammalian cells. Cell 1997;91:511-20.
17. Frankel G, Lider O, Hershkonz R, Mould AP, Kachalsky SG, Candy DCA, et al. The cell-binding domain of intimin from enteropathogenic *Escherichia coli* binds to beta-1 integrins. J Biol Chem 1996;271:20359-64.
18. Clark MA, Hirst BH, Jepson MA. M-cell surface beta-1 integrin expression and invasin-mediated targeting of *Yersinia pseudotuberculosis* to mouse Peyer's patch M cells. Infect Immun 1998;1237-43.
19. Finlay BB, Rosenshine I, Donnenberg MS, Kaper JB. Cytoskeletal composition of attaching and effacing lesions associated with enteropathogenic *Escherichia coli* adherence to HeLa cells. Infect Immun 1992;60:2541-3.
20. Sanger JM, Chang R, Ashton F, Kaper JB, Sanger JW. Novel form of actin-based motility transports bacteria on the surfaces of infected cells. Cell Motil Cytoskeleton 1996;34:279-87.
21. Knutton S, Baldwin T, Williams PH, McNeish AS. Actin accumulation at sites of bacterial adhesion to tissue culture cells: basis of a new diagnostic test for enteropathogenic and enterohemorrhagic *Escherichia coli*. Infect Immun 1989;57:1290-8.
22. Baldwin TJ, Ward W, Aitken A, Knutton S, Williams PH. Elevation of intracellular free calcium levels in HEp-2 cells infected with enteropathogenic *Escherichia coli*. Infect Immun 1991;59:1599-604.
23. Baldwin TJ, Lee-Delaunay MB, Knutton S, Williams PH. Calcium-calmodulin dependence of actin accretion and lethality in cultured HEp-2 cells infected with enteropathogenic *Escherichia coli*. Infect Immun 1993;61:760-3.
24. Matsudaira PT, Burgess DR. Structure and function of the brush-border cytoskeleton. Cold Spring Harb Symp Quant Biol 1982;46:845-54.
25. Dytoc M, Fedorko L, Sherman PM. Signal transduction in human epithelial cells infected with attaching and effacing *Escherichia coli* in vitro. Gastroenterology 1994;106:1150-61.
26. Foubister V, Rosenshine I, Finlay BB. A diarrheal pathogen, enteropathogenic *Escherichia coli* (EPEC), triggers a flux of inositol phosphates in infected epithelial cells. J Exp Med 1994;179:993-8.
27. Kenny B, Finlay BB. Intimin-dependent binding of enteropathogenic *Escherichia coli* to host cells triggers novel signaling events, including tyrosine phosphorylation of phospholipase C-gamma1. Infect Immun 1997;65:2528-36.
28. Crane JK, Oh JS. Activation of host cell protein kinase C by enteropathogenic *Escherichia coli*. Infect Immun 1997;3277-85.
29. Ben-Ami G, Ozeri V, Hanski E, Hofmann F, Aktories K, Hahn KM, et al. Agents that inhibit Rho, Rac, and Cdc42 do not block formation of actin pedestals in HeLa cells infected with enteropathogenic *Escherichia coli*. Infect Immun 1998;66:1755-8.
30. Takeuchi A. Electron microscope studies of experimental *Salmonella* infection. I. Penetration into the intestinal epithelium by *Salmonella typhimurium*. Am J Pathol 1967;50:109-36.
31. Finlay BB, Ruschkowski S, Dedhar S. Cytoskeletal rearrangements accompanying *Salmonella* entry into epithelial cells. J Cell Sci 1991;99:283-96.
32. Garcia del Portillo F, Finlay BB. *Salmonella* invasion of nonphagocytic cells induces formation of macropinosomes in the host cell. Infect Immun 1994;62:4641-5.
33. Francis CL, Ryan TA, Jones BD, Smith SJ, Falkow S. Ruffles induced by *Salmonella* and other stimuli direct macropinocytosis of bacteria. Nature 1993;364:639-42.
34. Galan JE. Molecular and cellular bases of *Salmonella* entry into host cells. Curr Top Microbiol Immunol 1996;209:43-60.
35. Ginocchio CC, Olmsted SB, Wells CL, Galan JE. Contact with epithelial cells induces the formation of surface appendages on *Salmonella typhimurium*. Cell 1994;76:717-24.
36. Zierler MK, Galan JE. Contact with cultured epithelial cells stimulates secretion of *Salmonella typhimurium* invasion protein InvJ. Infect Immun 1995;63:4024-8.
37. Fu Y, Galan JE. The *Salmonella typhimurium* tyrosine phosphatase SptP is translocated into host cells and disrupts the actin cytoskeleton. Mol Microbiol 1998;27:359-68.
38. Kaniga K, Uralil J, Bliska JB, Galan JE. A secreted tyrosine phosphatase with modular effector domains encoded by the bacterial pathogen *Salmonella typhimurium*. Mol Microbiol 1996;21:633-41.
39. Hardt WD, Chen LM, Schuebel KE, Bustelo XR, Galan JE. *S. typhimurium* encodes an activator of Rho GTPases that induces membrane ruffling and nuclear responses in host cells. Cell 1998;93:815-26.
40. Hardt WD, Urlaub H, Galan JE. A substrate of the centisome 63 type III protein secretion system of *Salmonella typhimurium* is encoded by a cryptic bacteriophage. Proc Natl Acad Sci U S A 1998;95:2574-9.
41. Chen LM, Hobbie S, Galan JE. Requirement of CDC42 for *Salmonella*-induced cytoskeletal and nuclear responses. Science 1996;274:2115-8.
42. Ruschkowski S, Rosenshine I, Finlay BB. *Salmonella typhimurium* induces an inositol phosphate flux in infected epithelial cells. FEMS Microbiol Lett 1992;74:121-6.
43. Adam T, Arpin M, Prevost MC, Gounon P, Sansonetti PJ. Cytoskeletal rearrangements and the functional role of T-plastin during entry of *Shigella flexneri* into HeLa cells. J Cell Biol 1995;129:367-81.
44. Clerc P, Sansonetti PJ. Entry of *Shigella flexneri* into HeLa cells: evidence for directed phagocytosis involving actin polymerization and myosin accumulation. Infect Immun 1987;55:2681-8.

45. Sansonetti PJ, Ryter A, Clerc P, Maruelli AT, Mounier J. Multiplication of *Shigella flexneri* within HeLa cells: lysis of the phagocytic vacuole and plasmid-mediated contact hemolysis. Infect Immun 1986;51:461-9.
46. Bernardini ML, Mounier J, d'Hauteville H, Coquis-Rondon M, Sansonetti PJ. Identification of icsA, a plasmid locus of *Shigella flexneri* that governs bacterial intra- and intercellular spread through interaction with F-actin. Proc Natl Acad Sci U S A 1989;86:3867-71.
47. Ogawa H, Nakamura A, Nakaya R. Cinemicrographic study of tissue cell cultures infected with *Shigella flexneri*. Jpn J Med Sci Biol 1968;21:259-73.
48. Zeile WL, Purich DL, Southwick FS. Recognition of two classes of oligoproline sequences in profilin-mediated acceleration of actin-based *Shigella* motility. J Cell Biol 1996;133:49-59.
49. Kadurugamuwa JL, Rohde M, Wehland J, Timmis KN. Intercellular spread of *Shigella flexneri* through a monolayer mediated by membranous protrusions and associated with reorganization of the cytoskeletal protein vinculin. Infect Immun 1991;59:3463-71.
50. Allaoui A, Mounier J, Prevost MC, Sansonetti PJ, Parsot C. *icsB*: a *Shigella flexneri* virulence gene necessary for the lysis of protrusions during intercellular spread. Mol Microbiol 1992;6:1605-16.
51. Lett MC, Saskawa C, Okada N, Sakai T, Makino S, Yamada M, et al. *virG*, a plasmid-coded virulence gene of *Shigella flexneri*: identification of the virG protein and determination of the complete coding sequence. J Bacteriol 1989;171:353-9.
52. Makino S, Sasakawa C, Kamata K, Kurata T, Yoshikawa M. A genetic determinant required for continuous reinfection of adjacent cells on large plasmid in *S. flexneri* 2a. Cell 1986;46:551-5.
53. Goldberg MB, Barzu O, Parsot C, Sansonetti PJ. Unipolar localization and ATPase activity of IcsA, a *Shigella flexneri* protein involved in intracellular movement. Infectious Agents Dispatch 1993;2:210-1.
54. Goldberg MB, Theriot JA. *Shigella flexneri* surface protein IcsA is sufficient to direct actin-based motility. Proc Natl Acad Sci U S A 1995;92:6572-6.
55. Kocks C, Marchand JB, Gouin E, d'Hauteville H, Sansonetti PJ. The unrelated surface proteins ActA of *Listeria monocytogenes* and IcsA of *Shigella flexneri* are sufficient to confer actin-based motility on *Listeria innocua* and *Escherichia coli* respectively. Mol Microbiol 1995;18:413-23.
56. Egile C, d'Hauteville H, Parsot C, Sansonetti PJ. SopA, the outer membrane protease responsible for polar localization of IcsA in *Shigella flexneri*. Mol Microbiol 1997;23:1063-73.
57. Shere KD, Sallustio S, Manessis A, d'Aversa TG, Goldberg MB. Disruption of IcsP, the major *Shigella* protease that cleaves IcsA, accelerates actin-based motility. Mol Microbiol 1997;25:451-62.
58. d'Hauteville H, Dufourcq Lagelouse R, Nato F, Sansonetti PJ. Lack of cleavage of IcsA in *Shigella flexneri* causes aberrant movement and allows demonstration of a cross-reactive eukaryotic protein. Infect Immun 1996;64:511-7.
59. Prevost MC, Lesourd M, Arpin M, Vernel F, Mounier J, Hellio R, et al. Unipolar reorganization of F-actin layer at bacterial division and bundling of actin filaments by plastin correlate with movement of *Shigella flexneri* within HeLa cells. Infect Immun 1992;60:4088-99.
60. Chakraborty T, Ebel F, Domann E, Niebuhr K, Gerstel B, Pistor S, et al. A focal adhesion factor directly linking intracellularly motile *Listeria monocytogenes* and *Listeria ivanovii* to the actin-based cytoskeleton of mammalian cells. EMBO J 1995;14:1314-21.
61. Suzuki T, Saga S, Sasakawa C. Functional analysis of *Shigella* VirG domains essential for interaction with vinculin and actin-based motility. J Biol Chem 1996;271:21878-85.
62. Suzuki T, Miki H, Takenawa T, Sasakawa C. Neural Wiskott-Aldrich-syndrome protein is implicated in the actin-based motility of *Shigella flexneri*. EMBO J 1998;17:2767-76.
63. Laine RO, Zeile W, Kang F, Purich DL, Southwick FS. Vinculin proteolysis unmasks an ActA homolog for actin-based *Shigella* motility. J Cell Biol 1997;138:1255-64.
